# Supplementary material for: Phylogenetic variation in cortical layer II immature neuron reservoir of mammals
Source: eLife. 2020 Jul 21;9:e55456. doi: 10.7554/eLife.55456 (PMC7373429; doi:10.7554/eLife.55456)
Supplement: Supplementary file 1. [file elife-55456-supp1.docx]

**Supplementary file 1.** Animals used in this study.

(a) Neuroscience Institute Cavalieri Ottolenghi (NICO); (b) School of Biological and Chemical Sciences, Queen Mary University of London, London; (c) Institute of Anatomy, University of Zurich; (d) INRA research center, Nouzilly, France; (e) University of Teramo (slaughter house); (f) Department of Comparative Biomedicine and Food Science, University of Padova; (g) National Chimpanzee Brain Resource, USA (www.chimpanzeebrain.org, supported by NIH grant NS092988). PMI: postmortem interval; CA, carotid artery; IC, intra-cardiac; PFA, paraformaldehyde solution. **^¶^** Only used for qualitative analysis.

| **Species** | **Source** | **Specimens** | **Age** | **Fixation** | **Fixative** | **PMI** |
| --- | --- | --- | --- | --- | --- | --- |
| **Mouse** | (a) | 4 | 10 days | Immersion | 4% PFA | A few minutes |
|  |  |  | 3 months | Perfusion (IC) |  | None |
|  |  |  | 6 months |  |  |  |
|  |  |  | 12 months |  |  |  |
| **NMR** | (b) |  | 2 months | Immersion |  | A few minutes |
|  |  |  | 2 years | Perfusion (IC) |  | None |
|  |  |  | 10 years |  |  |  |
| **WE bat** | (c) |  | Adults | Perfusion (IC) | 4% PAF  15% picric acid |  |
| **SC bat** | (c) |  | Adults | Perfusion (IC) |  |  |
| **Marmoset** | (c) |  | Adults | Immersion |  | 1 hour |
| **Rabbit** | (a) |  | 3 months | Perfusion (IC) | 4% PFA | None |
|  |  |  | 3 years |  |  |  |
| **Fox** | (c) |  | 7-8 months | Perfusion (CA) |  |  |
| **Sheep** | (d) |  | 4 months | Perfusion (CA) |  |  |
|  |  |  | 2 years |  |  |  |
|  | (e) |  | 8 – 10 years | Immersion | 10% formalin | 20 minutes |
| **Cat** | (f) |  | 1,5 years | Immersion | 4% formalin | Less than 1 hour |
|  |  |  | > 2 years |  |  |  |
| **Chimpanzee** | (g) |  | 19 – 27 years | Immersion | 10% formalin | Less than 14 hour |
|  |  |  | 40 – 48 years |  |  |  |
| **Sengi ^¶^** | (c) | 2 | 9 – 10 months | Perfusion (IC) | 4% PAF  15% picric acid | None |
| **Horse ^¶^** | (f) |  | Adults | Immersion | 4% formalin | Less than 20 minutes |
