# Supplementary material for: Phylogenetic variation in cortical layer II immature neuron reservoir of mammals
Source: eLife. 2020 Jul 21;9:e55456. doi: 10.7554/eLife.55456 (PMC7373429; doi:10.7554/eLife.55456)
Supplement: Supplementary file 2. — Species with lissencephalic, small-brains are shown in regular font and species with gyrencephalic, large-brains are shown in italics. [file elife-55456-supp2.docx]

**Supplementary file 2.** Main information on the animal species considered in this study. Species with more lissencephalic, small-brains are shown in regular font and species with more gyrencephalic, large-brains are shown in italics.

| **Species** | **Brain weight (g)**  (Zilles et al., 2013) | **Gyrification index**  (Zilles et al., 2013) | **Encephalization quotient**  (Roth and Dicke, 2005) | **Lifespan**  (Myers et al., 2019) | **Food habits**  (Myers et al., 2019) | **Habitat**  (Myers et al., 2019) |
| --- | --- | --- | --- | --- | --- | --- |
| **Mouse** | 0.6 | 1.03 | 0.5 | 1.5 y | Omnivorous | Terrestrial: forest |
| **NMR** | 0.5 | - | - | 30 y | Herbivorous | Terrestrial: savanna, grassland |
| **WE bat** | 1.75 | - | - | 10 y | Frugivorous | Terrestrial: rainforest |
| **SC bat** | 3.5 | - | - | 15-22 y | Herbivorous | Terrestrial: savanna, grassland, forest |
| **Marmoset** | 8.5 | 1.18 | 1.7 | 10-16 y | Herbivorous | Terrestrial: rainforest |
| **Rabbit** | 12 | 1.15 | 0.4 | 9 y | Herbivours | Terrestrial: savanna, grassland, forest, urban |
| ***Cat*** | 30 | 1.60 | 1.0 | 15 y | Carnivorous | Terrestrial: cosmopolitan |
| ***Fox*** | *45* | *2.01* | *1.6* | *12-15 y* | *Omnivorous* | *Terrestrial: savanna, grassland, forest, mountains, urban* |
| ***Sheep*** | *120* | *2.29* | *0.8* | *22.8 y* | *Herbivorous* | *Terrestrial: savanna, grassland, forest, mountains* |
| ***Chimpanzee*** | *384* | *2.31* | *2.2-2.5* | *60 y* | *Omnivorous* | *Terrestrial: savanna, grassland, forest* |
| **Sengi** | 1 | - | - | 1.5-6 y | Carnivorous | Terrestrial: scrub forest |
| ***Horse*** | *532* | *2.80* | *0.9* | *25-30 y* | *Herbivorous* | *Terrestrial: tundra, savanna, grassland, forest* |
