# Supplementary material for: Phylogenetic variation in cortical layer II immature neuron reservoir of mammals
Source: eLife. 2020 Jul 21;9:e55456. doi: 10.7554/eLife.55456 (PMC7373429; doi:10.7554/eLife.55456)
Supplement: Supplementary file 3. [file elife-55456-supp3.docx]

**Supplementary File 3.** Estimation of the neocortex surface area (calculated by using the median length of the layer II perimeter multiplied for 40 µm - thickness of sections - for the number of sections of the entire hemisphere).

| **SPECIES** | **Layer II perimeter**  **(mm)** | **Layer II perimeter for section thickness (40 µm)**  **(mm^2^)** | **Number of sections per brain (hemisphere)** | **Estimation of Neocortex surface area (mm^2^)** |
| --- | --- | --- | --- | --- |
| **Mouse** | 7.1 | 0.28 | 225 | **64** |
| **NMR** | 4.3 | 0.17 | 225 | **77** |
| **WE bat** | 12.4 | 0.5 | 200 | **99** |
| **SC bat** | 20.8 | 0.83 | 500 | **416** |
| **Marmoset** | 25.4 | 1.02 | 875 | **889** |
| **Rabbit** | 23.4 | 0.94 | 750 | **702** |
| **Fox** | 88.8 | 3.55 | 1625 | **5 772** |
| **Sheep** | 104.1 | 4.16 | 1625 | **6 767** |
| **Cat** | 64 | 2.56 | 1125 | **2 880** |
| **Chimpanzee** | 180.7 | 7.23 | 2750 | **19 877** |
