# Supplementary material for: Phylogenetic variation in cortical layer II immature neuron reservoir of mammals
Source: eLife. 2020 Jul 21;9:e55456. doi: 10.7554/eLife.55456 (PMC7373429; doi:10.7554/eLife.55456)
Supplement: Supplementary file 4. — Immature neurons prefer large brains [file elife-55456-supp4.docx]

**Supplementary File 4.** Estimation of the total number of DCX+ cells in layer II of the neocortex (calculated by multiplying the median number of DCX+ cells per section for the number of sections).

| **Animal ID** | **Mouse** | **NMR** | **WE bat** | **SC bat** | **Marmoset** | **Rabbit** | **Fox** | **Sheep** | **Cat** | **Chimpanzee** |
| --- | --- | --- | --- | --- | --- | --- | --- | --- | --- | --- |
| #1 | 0 | 1 | 14 | 5.5 | 26.5 | 69 | 450 | 1073 | 589 | 772 |
| #2 | 0 | 0 | 0 | 3 | 8.5 | 132 | 2021 | 968.5 | 1307 | 645 |
| #3 | 0.5 | 1.5 | 0 | 3 | 9 | 190.5 | 838 | 1099 | 503 | 898.5 |
| #4 | 0 | 0 | 1 | 7.5 | 7 | 79.5 | 462 | 809 | 510 | 847.5 |
| #5 | 0 | 0 |  |  |  |  |  | 258 | 1229.5 | 1050.5 |
| #6 | 0 | 0 |  |  |  |  |  | 554.5 | 888 | 745 |
| #7 | 0 | 0 |  |  |  |  |  | 145 | 1226 | 486 |
| #8 | 0 | 0 |  |  |  |  |  | 211.5 | 1286.5 | 759.5 |
| #9 | 0 |  |  |  |  |  |  |  |  |  |
| #10 | 0 |  |  |  |  |  |  |  |  |  |
| #11 | 0 |  |  |  |  |  |  |  |  |  |
| #12 | 0 |  |  |  |  |  |  |  |  |  |
| **DCX+ cells per section (Neocortex)** | 0 | 0 | 0.5 | 4.3 | 8.8 | 105.8 | 650 | 681.8 | 1057 | 765.8 |
| **Sections (40 μm thick)**  **per brain** | 225 | 225 | 200 | 500 | 875 | 750 | 1625 | 1625 | 1125 | 2750 |
| **Estimation of the number of DCX+ cells in Neocortex** | **0** | **0** | **100** | **2 125** | **7 656** | **79 313** | **1 056 250** | **1 107 844** | **1 189 125** | **2 105 813** |
